# Influence of the Printing Angle and Load Direction on Flexure Strength in 3D Printed Materials for Provisional Dental Restorations

**DOI:** 10.3390/ma14123376

**Published:** 2021-06-18

**Authors:** Paula Derban, Romeo Negrea, Mihai Rominu, Liviu Marsavina

**Affiliations:** 1 Department of Prosthetic Restorations on Implants, University of Medicine and Pharmacy Victor Babes of Timisoara, Blv. Revoluţiei, Nr. 9, 300041 Timişoara, Romania; paula.derban@umft.ro; 2 Department of Mathematics, University Politehnica of Timisoara, Pta. Victoriei, Nr. 2, 300006 Timisoara, Romania; romeo.negrea@upt.ro; 3 Department of Prosthesis Technology and Dental Materials, University of Medicine and Pharmacy Victor Babes of Timisoara, Blv. Revoluţiei, Nr. 9, 300041 Timişoara, Romania; rominu.mihai@umft.ro; 4 Department of Mechanics and Strength of Materials, University Politehnica of Timisoara, Mihai Viteazu Avenue, Nr. 1, 300222 Timisoara, Romania

**Keywords:** 3D printing, printing angle, load direction, provisional dental restorations, flexure strength

## Abstract

The CAD/CAM techniques, especially additive manufacturing such as 3D printing, constitute an ever-growing part of obtaining different dental appliances and restorations. Of these, provisional restorations are of frequent use in daily dental practice and are the object of this study. Masticatory and parafunctional forces determine flexure on these prostheses. This study investigates the influence of the printing angle and loading direction of the applied force on the flexure strength of two commercially available printable resins—Detax Freeprint Temp and Nextdent MFH Vertex dental. Ten rectangular beam specimens printed at the angle of 0, 45 and 90 degrees were fabricated of each of these materials, with an addition of 10 at 0 degrees for the investigation of the load direction. Three-point bending tests were performed in a universal testing machine. Flexure strength, strain at break and Young’s modulus were determined and a statistical analysis was performed on the obtained data. According to the statistical analysis, the flexural strength has a significance dependence with respect to degrees of orientation, for both investigated materials.

## 1. Introduction

In recent years the development of CAD/CAM (computer aided design/computer aided manufacturing) techniques using subtractive and additive manufacturing have greatly influenced many fields of work. The already established and widely used subtractive method (milling of blocks or disk cutting) faces a new competitor with the rise of a growing range of 3D printing techniques and printable polymers [1].

Restorative dentistry is an area that reaps the benefits of this development. Stereolithography (SLA) and the deriving digital light processing (DLP) technique are the most common in dental practice. A review on processes and mechanical models of polymers obtained using laser-based additive manufacturing was presented by Brighenti et al. [2].

The influence of manufacturing parameters, including printing directions and orientations, layer thickness, infill type, on mechanical properties, was investigated for different laser-based printing techniques [3,4].

3D printing is nowadays employed in the fabrication of surgical guides, custom trays [5] and diagnostic models [6], generally using polymers that do not need to be approved for intraoral use. Of interest to us are not these, but Class IIa CE-certified FDA-approved materials that are printable polymers approved for medium term intra-oral use, employed in the fabrication of provisional restorations.

Provisional fixed restorations have adopted this technique in their manufacturing process due to its advantages over more commonly used traditional direct and indirect means of fabrication: it requires less laboratory procedures [7] and is more economical as it involves less waste and wear of laboratory burs and other rotary tools and less waste of material [8]. This kind of restoration replaces the lost tooth material until the completion of the definitive prosthesis.

It needs to provide acceptable esthetics, occlusal support, alignment preservation of the prepared teeth [9] and be an aiding tool in diagnostic set-ups preceding the final restorations.

An important role of these provisional restorations is also to protect the prepared tooth [10] from physical factors, such as the forces that occur in the oral cavity during its physiological (and possible pathological) functions. Mastication is a complex biomechanical process characterized by the crushing and trituration of food by the teeth, helped by the mobilizing muscles of the mandible and some surrounding structures (tongue, lips, cheeks). Teeth play the main role in mastication, a part that is usually taken over by different prosthetic restorations, after tooth loss [11]. Other important forces that can appear in the oral cavity and exert a strain on teeth and prosthetic restorations are parafunctions, for example, in bruxism. Bruxism is a repetitive jaw-muscle activity characterized by clenching or grinding of the teeth and/or by bracing or thrusting of the mandible [12,13]. All these types of forces affect any restorations in the oral cavity. It is generally presumed that flexure strength is the main indicator of the mechanical response of a restorative material. Therefore, one of the most important properties to investigate is flexure strength.

In this study, two commercially available 3D printing materials, NEXTDENT MFH Vertex dental [14] and DETAX Freeprint Temp [15], will be evaluated regarding their flexure strength and flexure modulus. The purpose is to determine the influence of the printing parameters: angle and load direction, on the mentioned mechanical properties of the considered materials.

## 2. Materials and Methods

The chemical composition of the employed materials is generally undisclosed by the producers; therefore, we can only cite some of their content from the available literature. Nextdent C & B MFH is a microfilled commercially available provisional crown and bridge printable material (Nextdent C & B, vertex dental, Soesterberg, The Netherlands) with a matrix containing methacrylic oligomers and phosphine oxides [14]. Detax freeprint temp is a printable material containing 45–60 wt%, and is a monomer based on acrylic esters for 3D manufacturing of provisional crowns and bridges [15].

**Figure 1:**
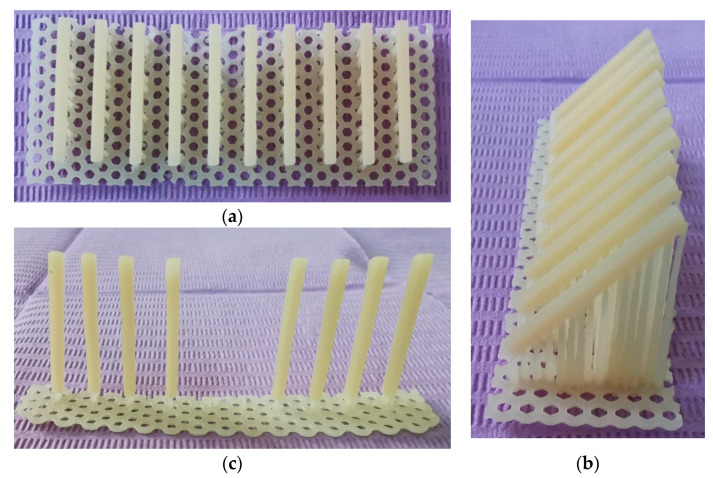
The printed specimens made of DETAX on three different directions: 0, 45 and 90 degrees. (**a**) 0°; (**b**) 45°; (**c**) 90°.

Ten parallelepiped shaped specimens at the printing angle 0, 45 and 90 of each of the two materials, DETAX Freeprint Temp (Detax Gmbh & Co KG, Ettlingen, Germany) and NEXTDENT C & B MFH (Vertex Dental B.V., Soesterberg, The Netherlands), were fabricated with a 50-microns layer thickness. An additional batch of 10 specimens of each material were printed at the angle of 0 to test the influence of the load direction in relation to the printing layers (Figure 1).

The specimens, selected according to the ADA-ANSI specification no. 27 [16] are 25 mm/2 mm/2 mm. The specimens were designed using the CAD software and computer file STL format was used to allow fabrication in a SheraPrint D30 printer (Shera Material Technology Gmbh, Lemforde, Germany).

All the specimens were then subjected to a postcuring treatment, according to the producer’s indications: in ethyl alcohol, in an ultrasonic bath three consecutive times, thoroughly dried under air pressure in between and postcured according to the manufacturer’s instructions in an Otoflash (Voco Gmbh, Cuxhaven, Germany) postcuring unit under nitrogen gas.

**Figure 2:**
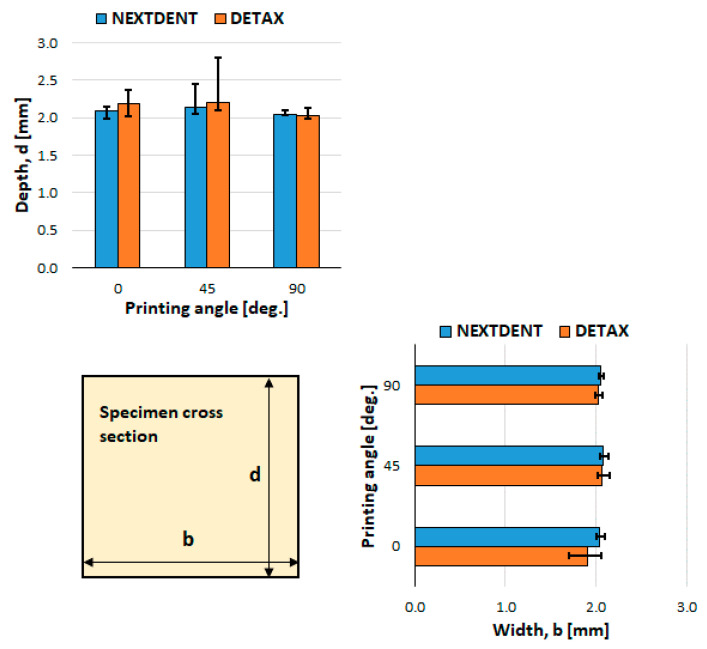
The specimens’ cross section, average and scatter (maximum/minimum measured—designed dimension) of b and d dimensions.

The specimens were measured after curing. A summary of the specimens’ average and scatter dimensions is presented in Figure 2. It could be observed that the 90° printing direction gives the more accurate dimensions for both b and d, while for the 45° printing direction the highest errors were obtained comparing with designed dimensions b = d = 2 mm. However, the flexure properties were determined on real specimen dimensions, measured with a caliper with 0.01 mm precision.

**Figure 3:**
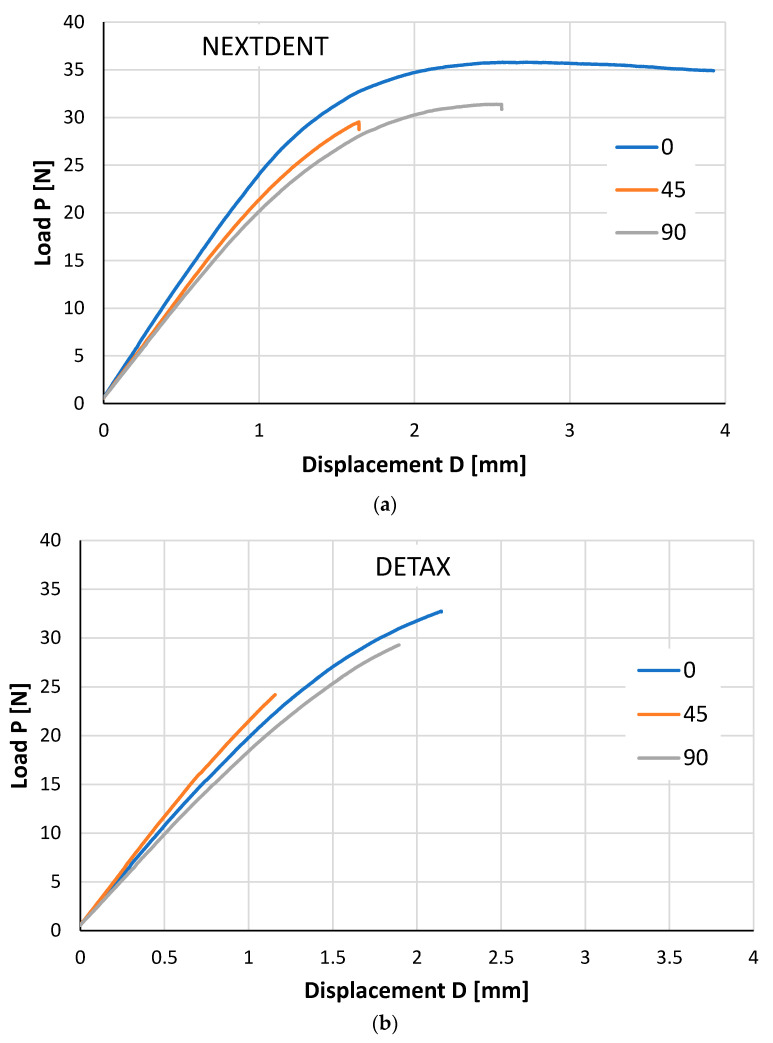
Typical load–displacement curves for different printing directions. (**a**) NEXTDENT; (**b**) DETAX.

Specimens were tested according to ASTM D790-Standard test method for flexural properties of unreinforced and reinforced plastics and electrical insulating materials [17]. The specimens were loaded in a three-point bending grip mounted on a ZWICK ProLine Z005 (Ulm, Germany) universal testing machine, according to ASTM D790 [17]. The span between the two supports was L = 21 mm. Tests were performed at room temperature (22 °C) with a loading speed of 2 mm/min. Typical load–displacement curves recorded during the tests are shown in Figure 3 for the two investigated materials (a. NEXTDENT and b. DETAX). A brittle behavior of DETAX material was observed for all printing directions (Figure 3a). For NEXTDENT material only 45° printing direction highlights a brittle behavior, and for 90° and 0° printing orientation a ductile behavior was observed.

The flexural strength *σ****_f_*** was estimated with the maximum load *P_max_* according to ASTM D790 [17]:

*σ_f_* = 3*P_max_L*/(2*bd*^2^) (1)

With *b* width of the specimen and *d* depth of the specimen. The flexural strain **ε*_f_*** was calculated as [17]:

ε*_f_*= 6*Dd*/*L*^2^ (2)

With *D* maximum deflection at the center of the beam. The chord method was used to calculate the flexural modulus, [17]:

*E_f_* = (*σ_f_*_2_ − *σ_f_*_1_)/(ε*_f_*_2_ − ε*_f_*_1_) (3)

where *ε_f_*_1_ = 0.0005 (0.05%) and *_f_ε*_2_ = 0.0025 (0.25%), respectively, represent the strains at which the corresponding stresses *σ_f_*_1_ and *σ_f_*_2_ were determined.

**Figure 4:**
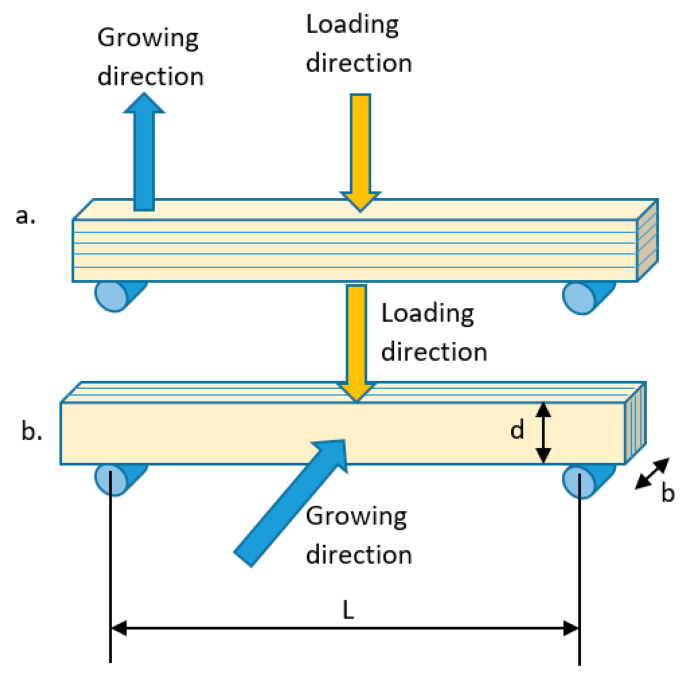
Loading directions (**a**) perpendicular to growing direction; (**b**) parallel to growing direction. b—specimen thickness, d—specimen depth, L—span between supports.

For the specimens printed at 0° angle, the load was applied perpendicular and parallel to the growth direction of the specimens (Figure 4).

## 3. Results and Statistical Analysis

The average values of 10 measurements for the main flexure properties determined experimentally are listed in Table 1 and Table 2 for the two considered materials.

It could be observed that higher values for Young’s modulus (with 18% for NEXTDENT and 20% for DETAX) and flexure strength (with 4.6% for NEXTEND and 26.5% for DETAX) were obtained when the load was applied parallel to growing direction. Similar values of Young’s modulus and flexure strength were obtained for 0° and 90° orientations for NEXTDENT material, while for 45° orientation both are lower (Table 1). For DETAX material the maximum values of Young’s modulus and flexure strength resulted in 90° orientation and the minimum ones for 45° (Table 2). The strains at break were higher for NEXTDENT compared to DETAX, except for the 0° orientation with parallel loading to growth direction.

The goal of statistical analysis was to compare these materials with respect to degrees of the printing angle. The first statistical hypothesis was: H0—all four ways have the same mean values of flexure strength and with the alternative hypothesis; H1—there exist differences between considered materials. The second statistical hypotheses were: H0—there exist two materials with the same (from statistical point of view) mean values of flexure strength (and of course with the alternative hypotheses, H1—there exist statistical differences between these two considered materials) for all pairs of these four types. The methods used are the one-way analysis of variance (ANOVA), Bartlett’s test of homogeneity of variances, pairwise *t*-tests with no assumption of equal variances, ANOVA test with no assumption of equal variances and a non-parametric alternative to one-way ANOVA–Kruskal–Wallis rank sum test, which can be used when ANOVA assumptions are not met.

It is known that boxplots are a standardized way of displaying the distribution of data based on a five-number summary (“minimum”, first quartile (Q1), median, third quartile (Q3), and “maximum”). The points from the dataset that are not between these “minimum” and “maximum” points are called outliers. The “minimum” is not necessarily the smallest number from the data. More precisely, we have the following: Minimum (Q0 or 0th percentile): the lowest data point excluding any outliers, and Maximum (Q4 or 100th percentile): the largest data point excluding any outliers. The outliers are values from the data that are greater than 1.5IQR + Q3, or, values that are smaller than 1.5IQR − Q1; IQR = Q3 − Q1 is called the “interquartile rage”. In all four statistical samples, we do not have outliers (i.e., the datasets are very good for a statistical analysis).

**Figure 5:**
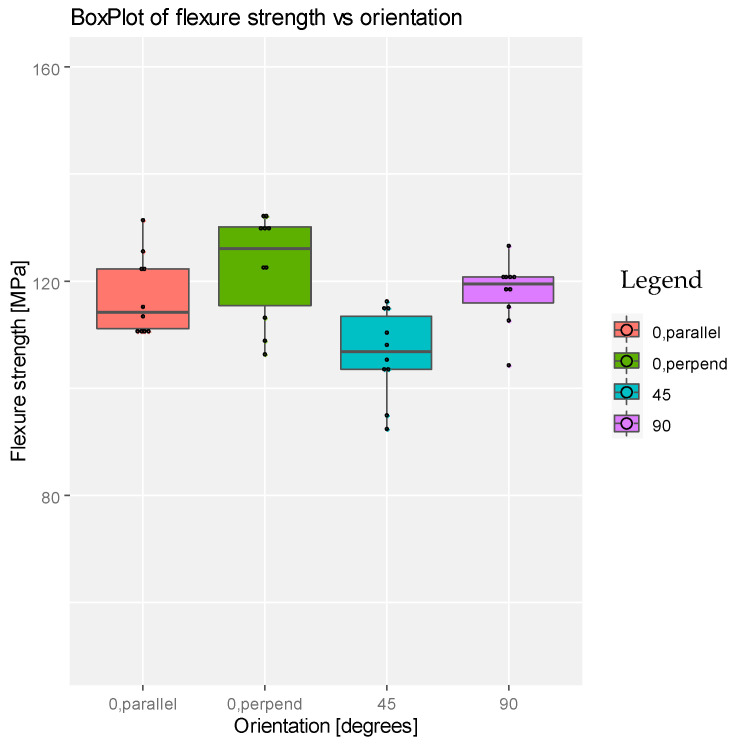
Boxplot representation for the flexure strength with respect to degrees of printing angle for the product NEXTDENT.

**Figure 6:**
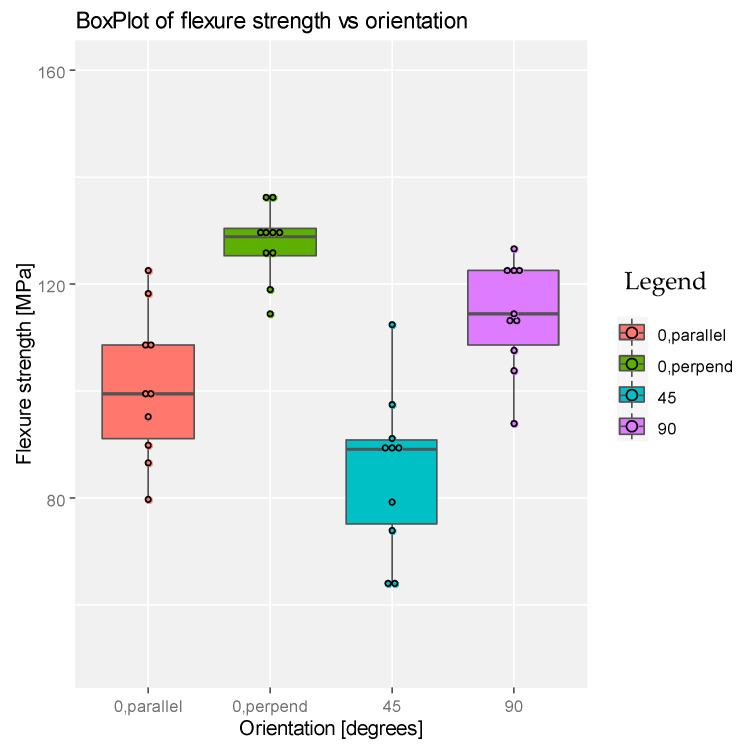
Boxplot representation for the flexure strength with respect to degrees of printing angle for the product DETAX.

For both dental products we observed (see Figure 5 and Figure 6) that the higher flexural strength is obtained at 0 degrees—perpendicular loading direction—and it seems to be not so good for specimens printed at 45 degrees. Also, both graphs show some differences between these four types of printing, for both dental products.

Also, a simple calculation of the mean value and the variance for each type suggested some difference between the ways of printing for both dental products (see Table 3 and Table 4).

After these simple and descriptive statistics, we implemented the methods of statistical inferences. The first method used was (classical one-way) ANOVA. The one-way analysis of variance (ANOVA), also known as one-factor ANOVA, is an extension of an independent two-samples *t*-test for comparing mean values in a situation where there are more than two groups (see [18]). In one-way ANOVA, the data is organized into several groups based on one single grouping variable (also called factor variable). We used the function res.aov (see [19]) from the R software (version 4.0.0 (2020-04-24)) We obtain, for both dental products (see Table 5 and Table 6), respectively:

where:

- The Df column displays the degrees of freedom for the independent variable (the number of levels in the variable minus 1), and the degrees of freedom for the residuals (the total number of observations minus one and minus the number of levels in the independent variables).
- The Sum Sq column displays the sum of squares (a.k.a. the total variation between the group means and the overall mean).
- The Mean Sq column is the mean of the sum of squares, calculated by dividing the sum of squares by the degrees of freedom for each parameter.
- The F-value column is the test statistic from the F test. This is the mean square of each independent variable divided by the mean square of the residuals. The larger the F value, the more likely it is that the variation caused by the independent variable is real and not due to chance.
- The Pr (>F) column is the *p*-value of the F-statistic. This shows how likely it is that the F-value calculated from the test would have occurred if the null hypothesis of no difference among group means were true.
- Signif. Codes for asterixs or points that can be observed in the column Pr (>F) (i.e., *p*-values column) are the following: “***“ means that *p*-value is less than 0.001, “**” means that *p*-value is between 0.01 and 0.001, “*” means that *p*-value is between 0.01 and 0.05, “.” means that *p*-value is between 0.05 and 0.1.

*p*-values of both variables are low (*p* < 0.001), so it appears that the way of printing for both dental products has a statistically significant impact on the flexure strength.

The ANOVA test assumes that the data are normally distributed and the variance across groups is homogeneous (see [18]). A normal distribution of the data as well as the homogeneity of variance can be checked with various diagnostic tests.

First, we checked the homogeneity of variance assumption. The residuals versus fits plot can be used to check the homogeneity of variances.

**Figure 7:**
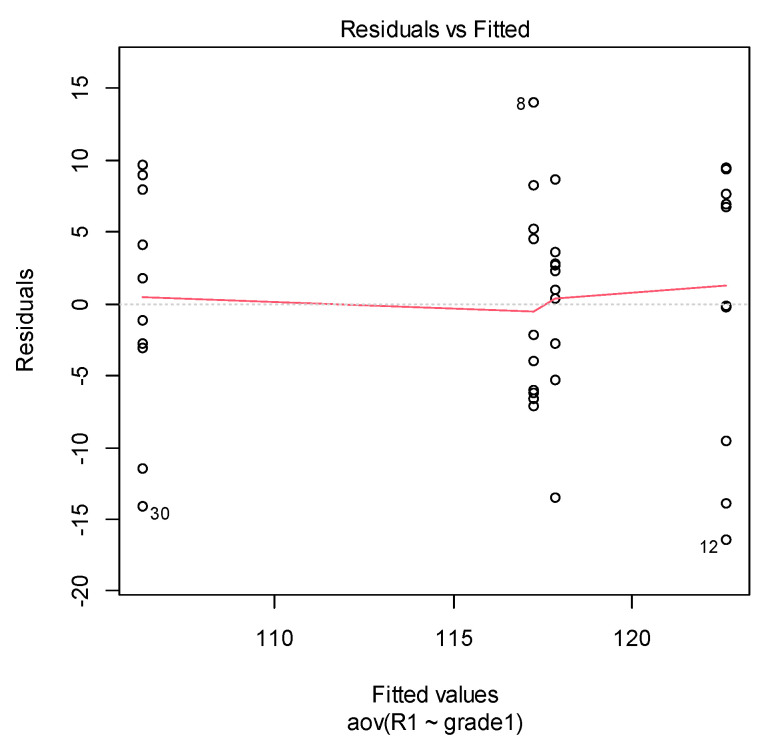
Residuals vs. fitted from ANOVA in NETXDENT material case.

**Figure 8:**
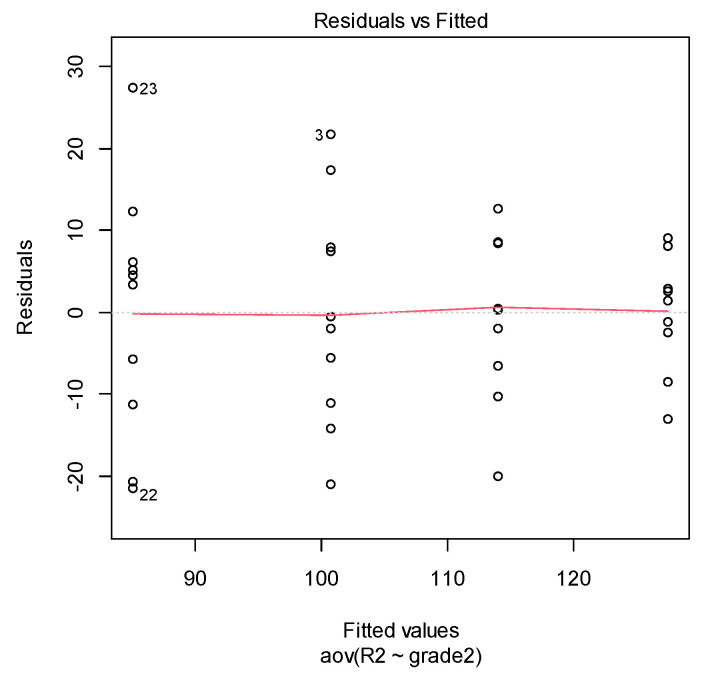
Residuals vs. fitted from ANOVA in DETAX material case.

If the residuals form an approximate horizontal line around the 0 line, then this indicates a homogeneity of error variance. In our cases (see Figure 7 and Figure 8), there is no evident relationship between residuals and fitted values (the mean of each groups). More exactly, both graphs show a non-homogeneity in variance (i.e., a heteroscedasticity).

Secondly, the normal distribution of the data can be checked with various diagnostic tests. The normal probability plot of residuals is used to check the assumption that the residuals are normally distributed. It should approximately follow a straight line.

**Figure 9:**
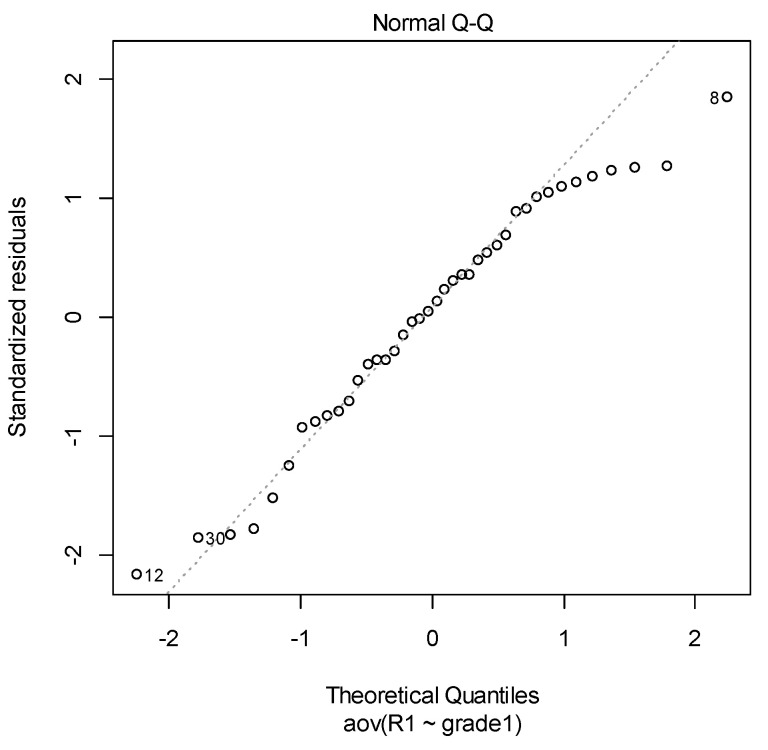
Q–Q plot from ANOVA in NEXTDENT case.

**Figure 10:**
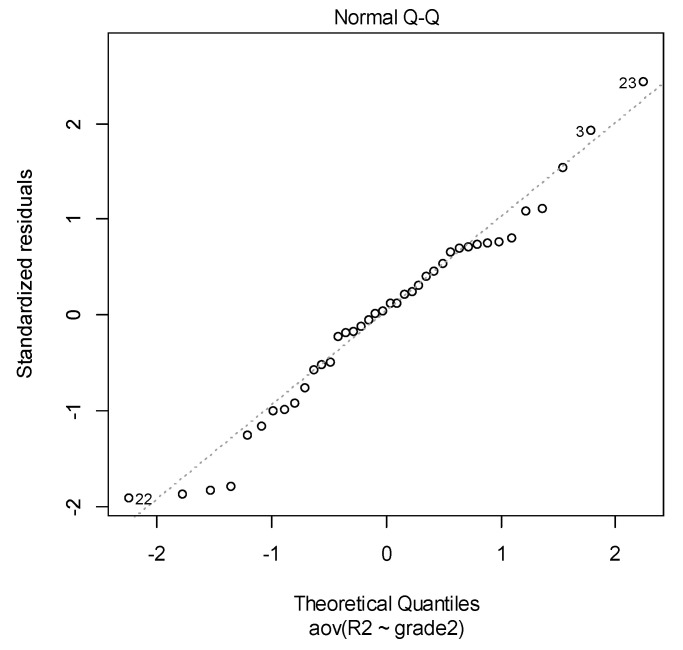
Q–Q plot from ANOVA in DETAX case.

For both considered materials graphs in Figure 9 and Figure 10 suggest that the hypothesis of normality is satisfied. Of course, the graphical way is not enough rigorous to sustain a statistical assumption. We used two statistical tests for a solid verification of these two assumptions.

More precisely, we used the Bartlett’s test to compare multiple variances. Statistical hypothesis, i.e., the null hypothesis is that all variances for all four orientation angles, for both dental products, are equal, and the alternative hypothesis is that at least two of them differ. Consequently, *p*-values less than 0.05 suggest that the variances are significantly different and that the homogeneity of variance assumption has been violated. Also, we used the bartlett.test function from R software (see [20]).

Both results (*p*-values < 0.05, see Table 7 and Table 8) means that homogeneity of variances (called homoscedasticity) assumption is not true.

Also, for the normality assumption we used the Shapiro–Wilk test on the ANOVA residuals (*p*-values > 0.05, see Table 9 and Table 10) which found no indication that normality is violated (we used the shapiro.test function from R software, see [21]).

In conclusion, one assumption for the ANOVA method has been violated. Therefore, other methods must be used for a good statistical analysis (see [22,23,24,25]). We will show some of these methods in the following.

First method is the so-called “Pairwise *t*-tests with no assumption of equal variances”. After a simple implementation of this test in R software, we found the following (see Table 11 and Table 12):

For the dental product NEXTDENT, the above table shows a high *p*-value for the comparison of the following ways of printing: “0 degree parallel with 90 degree”, i.e., both ways of printing product have almost the same (in mean value) flexure strength. For the rest cases *p*-value have low values, which suggested that it is false to sustain a similar behavior of flexure strength. For the dental product DETAX, all ways of printing product have different flexure strength (the *p*-values have very low values). We mention that the *p*-values were adjusted by the Benjamini–Hochberg method.

The second method it is an ANOVA method version obtained by relaxing the assumption of homogeneity of variance. We used the function oneway.test, ANOVA test with no assumption of equal variances (see [26]).

We observed that for both dental products (see Table 13 and Table 14), the *p*-values were lower than the significance level of 0.05 (*p*-values are 0.004171 and 0.0000005906, respectively), i.e., the null hypothesis (equality of all mean values of flexure strength for the four orientation angles) was rejected.

We mention that a non-parametric alternative to one-way ANOVA is the Kruskal–Wallis rank sum test, which can be used when ANOVA assumptions are not met (see [21,27]).

Also, the *p*-value of the test (see Table 15 and Table 16) suggests that we must reject the null hypothesis of the equality of the four mean values, for both dental product (*p*-values < 0.05).

In conclusion, the statistical analysis sustains the assumption that the flexure strength depends by the degrees of angle printing for both dental products. The coefficient of correlation between the flexure strength and the degrees of angle printing (−0.1892191 for NEXTDENT and −0.1170141 for DETAX) suggested a slow linear dependence between these variables.

## 4. Conclusions

The stereolithography (SLA) technique was employed to 3D printing of rectangular specimens using two plastic materials used for provisional dental restorations (NEXTDENT and DETAX as trade name). Three different growing directions were considered (0, 45 and 90 degrees) and for specimens printed at 0 degrees two loading directions were considered parallel and perpendicular with the printing plane.

Specimens were tested in three-point bending and Young’s modulus, flexural strength and strain at break were determined.

Higher values for Young’s modulus (with 18% for NEXTDENT and 20% for DETAX) and flexure strength (with 4.6% for NEXTEND and 26.5% for DETAX) were obtained when the load was applied parallel to growing direction, which is in agreement with [28]. Similar values of Young’s modulus and flexure strength were obtained for 0° and 90° orientations for NEXTDENT material, while for 45° orientation both are lower. For the DETAX material the maximum values of Young’s modulus and flexure strength resulted on 90° orientation and the minimum ones for 45°. The strains at break were higher for NEXTDENT compared to DETAX, showing a more brittle behavior of DETAX material, with one exception at 0° orientation and loading parallel to growing direction.

Considering the flexural strength, to determine the most important property for the provisional dental restorations a statistical analysis for flexural strength was employed.

A robust statistical analysis was performed, and two important conclusions can be drawn: the first conclusion is that the flexural strength has a significant dependence with respect to degrees of orientation, for both materials. The second conclusion expresses that for DETAX material the influence of orientation is much more evident than for NEXTDENT material.

An important technical aspect is the influence of the printing orientation on the dimensions of the resulted printed specimens. The most accurate dimensions were obtained for printing at 90° orientation, while the highest errors were obtained for 45° printing orientation. Similar results are presented for compressive strength in [29].

This study highlighted that printing orientation and applied loading direction influence the mechanical properties of printed specimens. The obtained results are of practical importance when designing and manufacturing provisional restorations using SLA technology.

## Figures and Tables

**Table 1:** Flexure test results for NEXTDENT.

Specimen Orientation (Degrees)	Loading Direction	Young Modulus (MPa)	Flexure Strength (MPa)	Strain at Break (%)
0	parallel	3284.79	122.61	6.19
0	perpendicular	2765.42	117.24	8.83
45	–	2610.48	106.35	5.46
90	–	2766.83	117.84	8.07

**Table 2:** Flexure test results for DETAX.

Specimen Orientation (Degrees)	Loading Direction	Young Modulus (MPa)	Flexure Strength (MPa)	Strain at Break (%)
0	parallel	2703.69	127.54	7.62
0	perpendicular	2253.38	100.76	6.45
45	–	2453.25	85.05	3.81
90	–	2542.17	113.98	5.55

**Table 3:** Mean value and variance for NEXTDENT material.

Statistical Parameter	0 Degree Parallel	0 Degree Perpendicular	45 Degrees	90 Degrees
Mean value	117.236	122.608	106.348	117.843
Variance	55.83274	97.75551	67.40968	36.41227

**Table 4:** Mean value and variance for DETAX material.

Statistical Parameter	0 Degree Parallel	0 Degree Perpendicular	45 Degrees	90 Degrees
Mean value	100.754	127.539	85.058	113.980
Variance	188.49760	46.21454	226.84195	103.64136

**Table 5:** ANOVA results of flexure strength for NEXTDENT material.

Material	Df	Sum Sq	Mean Sq	F Value	Pr (>F)
NEXTDENT	3	1418	472.5	7.342	0.000581 ***
Residuals	36	2317	64.4		
Significance codes:	0 ‘***’ 0.001 ‘**’ 0.01 ‘*’ 0.05 ‘.’ 0.1 ‘ ’ 1				

**Table 6:** ANOVA results of flexure strength for DETAX material.

Material	Df	Sum Sq	Mean Sq	F Value	Pr (>F)
DETAX	3	9909	3303	23.38	0.000001.42 ***
Residuals	36	5087	141	–	–
Significance codes:	0 ‘***’ 0.001 ‘**’ 0.01 ‘*’ 0.05 ‘.’ 0.1 ‘ ’ 1				

**Table 7:** Bartlett’s test for flexure strength for NEXTDENT material.

Bartlett’s test of homogeneity of variances		
data: R1 by grade1		
Bartlett’s K-squared = 2.1245,	df = 3,	*p*-value = 0.547

**Table 8:** Bartlett’s test for flexure strength for DETAX material.

Bartlett’s test of homogeneity of variances		
data: R2 by grade2		
Bartlett’s K-squared = 5.7283,	df = 3,	*p*-value = 0.1256

**Table 9:** Shapiro–Wilk test for flexure strength for NEXTDENT material.

Shapiro–Wilk normality test	
data: aov_residuals1	
W = 0.96326	*p*-value = 0.2161

**Table 10:** Shapiro–Wilk test for flexure strength for DETAX material.

Shapiro–Wilk normality test	
data: aov_residuals2	
W = 0.97589	*p*-value = 0.5405

**Table 11:** Mean value and variance for NEXTDENT material.

Pairwise comparisons using *t*-tests with pooled SD			
data: df1$R1 and df1$grade1			
–	0, parallel	0, perpend	45
0, perpend	0.21451	–	–
45	0.00890	0.00037	–
90	0.86659	0.23096	0.00851

*p* value adjustment method: BH.

**Table 12:** Mean value and variance for DETAX material.

Pairwise comparisons using *t*-tests with pooled SD			
data: df2$R2 and df2$grade2			
	0, parallel	0, perpend	45
0, perpend	0.00027	–	–
45	0.0083	0.00000001	–
90	0.0176	0.0176	0.000012

*p* value adjustment method: BH.

**Table 13:** One-way analysis of mean (not assuming equal variances) for NEXTDENT material.

One-way analysis of means (not assuming equal variances)			
data: R1 and grade1			
F = 6.0859	num df = 3.000	denom df = 19.719	*p*-value = 0.004171

**Table 14:** One-way analysis of mean (not assuming equal variances) for DETAX material.

One-way analysis of means (not assuming equal variances)			
data: R2 and grade2			
F = 26.075	num df = 3.000	denom df = 19.05	*p*-value = 0.0000005906

**Table 15:** Kruskal–Wallis rank sum test for NEXTDENT material.

Kruskal-Wallis rank sum test		
data: R1 by grade1		
Kruskal–Wallis chi-squared = 13.348	df = 3	*p*-value = 0.003942

**Table 16:** Kruskal–Wallis rank sum test for DETAX material.

Kruskal–Wallis rank sum test		
data: R2 by grade2		
Kruskal–Wallis chi-squared = 27.101	df = 3	*p*-value = 0.000005607

## Data Availability

Data sharing is not applicable.
